# Suppression of BRCA1 sensitizes cells to proteasome inhibitors

**DOI:** 10.1038/cddis.2014.537

**Published:** 2014-12-18

**Authors:** Y Gu, P Bouwman, D Greco, J Saarela, B Yadav, J Jonkers, S G Kuznetsov

**Affiliations:** 1 Institute for Molecular Medicine Finland (FIMM), University of Helsinki, PO Box 20, Helsinki 00014, Finland; 2 Division of Molecular Pathology and Cancer Genomics Centre, The Netherlands Cancer Institute, Amsterdam, The Netherlands; 3 Unit of Systems Toxicology, Finnish Institute of Occupational Health (FIOH), Helsinki, Finland

## Abstract

BRCA1 is a multifunctional protein best known for its role in DNA repair and association with breast and ovarian cancers. To uncover novel biologically significant molecular functions of BRCA1, we tested a panel of 198 approved and experimental drugs to inhibit growth of MDA-MB-231 breast cancer cells depleted for BRCA1 by siRNA. 26S proteasome inhibitors bortezomib and carfilzomib emerged as a new class of selective BRCA1-targeting agents. The effect was confirmed in HeLa and U2OS cancer cell lines using two independent siRNAs, and in mouse embryonic stem (ES) cells with inducible deletion of *Brca1*. Bortezomib treatment did not cause any increase in nuclear foci containing phosphorylated histone H2AX, and knockdown of BRCA2 did not entail sensitivity to bortezomib, suggesting that the DNA repair function of BRCA1 may not be directly involved. We found that a toxic effect of bortezomib on BRCA1-depleted cells is mostly due to deregulated cell cycle checkpoints mediated by RB1-E2F pathway and 53BP1. Similar to BRCA1, depletion of RB1 also conferred sensitivity to bortezomib, whereas suppression of E2F1 or 53BP1 together with BRCA1 reduced induction of apoptosis after bortezomib treatment. A gene expression microarray study identified additional genes activated by bortezomib treatment only in the context of inactivation of BRCA1 including a critical involvement of the ERN1-mediated unfolded protein response. Our data indicate that BRCA1 has a novel molecular function affecting cell cycle checkpoints in a manner dependent on the 26S proteasome activity.

*BRCA1* is an important tumor suppressor gene whose germ-line or somatic inactivation is implicated in a significant number of breast and ovarian cancers.^1^ Human BRCA1 encodes an 1863 amino-acid-long protein with a RING-finger domain at the N terminus and two BRCT domains located at the C terminus.^2, 3^ BRCT domains mediate interaction with phosphorylated proteins such as Abraxas, BACH1, CtIP and others involved in sensing DNA damage and assembly of the BRCA1-associated genome surveillance complex at sites of DNA breaks.^4^ The RING domain constitutively interacts with the BRCA1-associated RING domain protein (BARD1), forming a heterodimer having an E3 ubiquitin ligase activity.^5^ Ubiquitination of target proteins, including cell cycle or DNA repair-regulating proteins (e.g. CtIP (RBBP8), nucleophosmin (NPM1, B23), claspin (CLSPN) and others), occurs either at Lys48 residue of the ubiquitin leading to the 26S proteasome-mediated degradation of target proteins or at Lys6 or Lys63 having a trafficking and signaling role.^6^ A serine cluster coiled-coil domain spanning amino acids 1280–1524 contains multiple phosphorylation sites for ATM and ATR kinases activated by DNA damage.^7^ The same region also binds PALB2 protein linking BRCA1 to another major breast cancer predisposition gene *BRCA2.*^8^

The most prominent function of BRCA1 is associated with its role in repair of DNA damage, particularly of double-stranded DNA breaks (DSBs), one of the most severe types of DNA lesions.^9^ BRCA1 is recruited to sites of DNA damage via a series of phosphorylation and ubiquitination events, where it serves as a binding scaffold for other DNA repair proteins,^10, 11^ ubiquitinates claspin, cyclin B and CDC25C, triggering cell cycle arrest to allow time for repair,^12^ and facilitates BRCA2-mediated loading of RAD51 recombinase to enable the homologous recombination (HR) mechanism of DNA repair.^9^ In addition, BRCA1 may contribute to maintaining genome integrity by stabilizing the heterochromatin structure via ubiquitination of histone H2A.^13^ BRCA1 is also required for centrosome-dependent and -independent mitotic spindle formation, providing another route, by which loss of BRCA1 could promote chromosome instability and tumor formation.^14, 15^

Such a critical role of BRCA1 in DNA repair is exploited therapeutically. DNA-damaging agents, particularly DNA-crosslinking agents such as platinum-containing drugs, or ionizing radiation lead to the accumulation of DNA breaks requiring HR for repair and, therefore, are particularly toxic to BRCA1-deficient tumor cells.^16^ Pharmacological inhibitors of poly-(ADP-ribose) polymerases (PARPs) selectively kill BRCA1-deficient cells owing to defective HR, functioning as a back-up repair mechanism in the absence of the PARP-mediated repair of single-stranded DNA breaks.^17^ However, multiple mechanisms allow BRCA1-deficient cells to develop resistance to these drugs including elevated expression of the efflux transporters pumping the drugs out of the cell, secondary mutations restoring a functional BRCA1 protein and loss of 53BP1 protein, which counteracts BRCA1 and HR by blocking resection of DNA ends around the breaks (see Lord and Ashworth^18^ for the latest review). Therefore, additional efforts to identify small-molecule agents especially targeting BRCA1 functions unrelated to its DNA repair function are warranted.

Here we performed a high-throughput chemical screen of BRCA1-depleted MDA-MB-231 cells using a collection of 198 US Food and Drug Administration (FDA)-approved and experimental drugs. We found that 26S proteasome inhibitors were more toxic to BRCA1 knockdown than control cells. Response of BRCA1-deficient cells to bortezomib involved deregulation of the RB1-mediated cell cycle checkpoint, activation of a noncanonical ERN1-mediated unfolded protein response and 53BP1-related G2/M cell cycle arrest. Our results reveal novel aspects of BRCA1 function unrelated to DNA repair.

## Results

### Proteasome inhibitors selectively kill BRCA1-deficient cells

**Figure 1:**
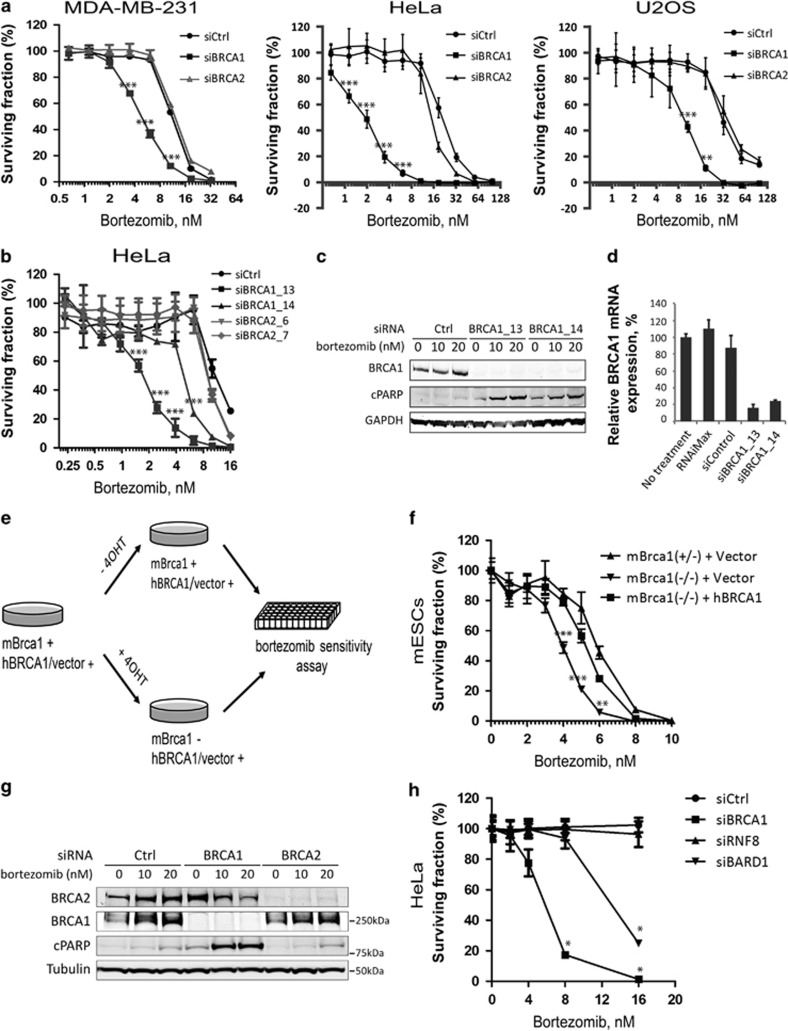
Proteasome inhibitors inhibit the growth of BRCA1- but not BRCA2-deficient cells. (**a**) Knockdown of BRCA1, but not BRCA2, sensitizes MDA-MB-231 (left), HeLa (middle) and U2OS (right) cells to a proteasome inhibitor bortezomib. Cell viability was measured in 96-well plates using CellTitre-Blue reagent to detect viable cells after 4 days of incubation with indicated doses of bortezomib. All treatments were performed in four replicas (*N*=4). (**b**) Same as (**a**) with two independent siRNAs for BRCA1 and BRCA2 in HeLa cells; *N*=4. (**c**) Western blot showing that the level of cleaved PARP (cPARP) correlates with efficiency of BRCA1 depletion at the mRNA level (**d**) and cell viability (**b**). (**d**) qRT-PCR demonstrating the efficiency of BRCA1 knockdown for two siRNAs in HeLa cells. (**e**) Experimental scheme for inducing genetic deletion of *Brca1* in mouse ES cells. (**f**) Loss of murine *Brca1* (mBrca1) sensitizes mouse ES cells to bortezomib, which is rescued by the expression of human BRCA1 (hBRCA1); *N*=3. (**g**) Western blot demonstrating siRNA-mediated knockdown of BRCA1/2 and induction of apoptosis measured by the amount of cPARP in HeLa cells treated with indicated doses of bortezomib for 20 h. (**h**) Similar to BRCA1, depletion of BARD1 (siBARD1), but not RNF8 (siRNF8), sensitizes HeLa cells to bortezomib; *N*=2. Error bars in (**a**, **b**, **d**, **f**, and **h**) indicate S.D. RNAiMAX, effect of the Lipofectamine RNAiMAX reagent on the expression of BRCA1; siBRCA1, siRNAs targeting BRCA1; siControl, non-targeting negative control siRNA

High-throughput small-molecule screening has been widely used to identify pharmacologically relevant compounds targeting cancer cells with specific genetic abnormalities. We used a locally available library of 198 FDA-approved drugs or targeted investigational drugs^19^ to investigate pharmacological vulnerabilities of human cells, in which expression of BRCA1 was transiently suppressed using an siRNA-mediated knockdown. The screen was performed on the breast cancer cell line MDA-MB-231, which carries several typical features of BRCA1-deficient breast cancers, for example, expression of basal epithelial markers, negativity for hormone receptors, and a high metastatic potential.^20^ Cells transfected with siRNA against BRCA1 (siBRCA1) or non-targeting control siRNA (siCtrl) were grown in the presence of chemical compounds at five different concentrations in 384-well plates for 3 days. Dose–response proliferation curves and corresponding metrics for all drugs are given in Supplementary Table S2. We found that two 26S proteasome inhibitors, bortezomib and carfilzomib, demonstrated a stronger growth-inhibitory effect on BRCA1-depleted cells compared with control cells (Supplementary Table S2). To verify this observation, we tested the sensitivity of MDA-MB-231 cells as well as two other commonly used cancer cell lines, HeLa and U2OS, to both drugs at 10 different doses in a 96-well plate format (Figure 1a and Supplementary. Figure 1a). The 50% inhibitory concentration (IC_50_) for all BRCA1-depleted cells was found to range between 2 and 10 nM for bortezomib and between 3 and 25 nM for carfilzomib. Importantly, depletion of BRCA1 conferred a minimum of 2.5- to 3-fold (MDA-MB-231 and U2OS) and a maximum of 8- to 10-fold (HeLa) sensitization to both drugs relative to control cells (Figure 1a). A second independent siRNA targeting BRCA1 (siBRCA1_14) also sensitized HeLa and U2OS cells to bortezomib, although about four times less efficiently based on IC_50_ values (Figure 1b). Similar differences between two siRNAs were also evident by higher amounts of cleaved PARP in cells treated with siBRCA1_13 (Figure 1c). Although both siRNAs appeared equally efficient at the protein level, siBRCA1_14 was markedly less efficient than siBRCA1_13 at the mRNA level (residual amount of BRCA1 mRNA was 24% for siBRCA1_14 *versus* 16% for siBRCA1_13; Figure 1d), which likely explains the differences in sensitization to bortezomib.

To further confirm the specificity of BRCA1-dependent sensitization to proteasome inhibitors and eliminate the possibility of an artifact caused by an off-target effect of the siRNA treatment, we used a mouse ES cell model with a conditional *Brca1* knockout.^21^ Here conditional *Brca1*-knockout ES cells carrying a human *BRCA1* cDNA expression cassette or an empty RMCE vector were treated with 4-hydroxytamoxifen (4-OHT) to induce deletion of *Brca1*, and then tested for sensitivity to bortezomib as illustrated (Figure 1e). Consistent with previous results, ES cells lacking *Brca1* expression were 1.5-fold more sensitive to bortezomib compared with heterozygous control cells (Figure 1f). Importantly, expression of human *BRCA1* cDNA restored sensitivity to bortezomib almost to the level of control cells (Figure 1f). Taken together, our data demonstrate that inhibition of *BRCA1* expression can indeed sensitize a range of mammalian cell types to proteasome inhibitors *in vitro*.

### Bortezomib does not induce nuclear *γ*H2AX foci and is not toxic for BRCA2-depleted cells

**Figure 2:**
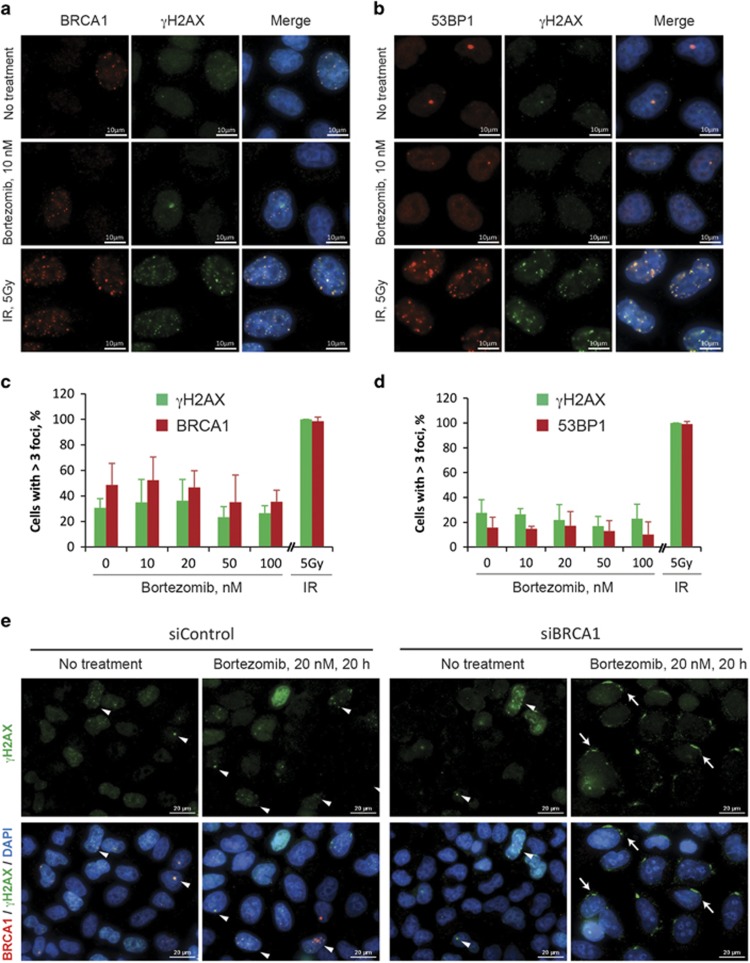
Bortezomib does not induce *γ*H2AX nuclear foci. HeLa cells with or without treatment with 10 nM bortezomib for 8 h were stained for BRCA1 and *γ*H2AX (**a**) or BRCA1 and 53BP1 nuclear foci (**b**). Treatment with 5 Gy ionizing radiation was used as a positive control. Note that bortezomib-treated cells do not show increased number of foci. (**c** and **d**) Quantification of the experiment exemplified in (**a**) and (**b**), respectively, showing the percentage of foci-positive nuclei. Five view fields containing 73–103 nuclei in total were quantified for each sample. Nuclei were considered positive if they contained over three nuclear foci. Error bars show S.E.M. Each treatment was performed in triplicates. (**e**) Treatment with bortezomib for 20 h does not induce *γ*H2AX nuclear foci even in BRCA1-depleted HeLa cells (siBRCA1). Arrows show perinuclear *γ*H2AX likely indicating induction of apoptosis in BRCA1-depleted cells treated with bortezomib. Arrowheads mark *γ*H2AX-positive nuclear foci. Scale bars=10 *μ*m

Bortezomib and other proteasome inhibitors are shown to delay or inhibit various stages of HR and the Fanconi anemia pathway.^22, 23^ We argued that, if sensitization to bortezomib is due to the role of BRCA1 in HR, knockdown of BRCA2, which is even more directly involved in HR, should produce the same effect. Surprisingly, cells depleted for BRCA2 using two independent siRNAs did not show any increase in sensitivity to either bortezomib or carfilzomib in any cell line tested, suggesting that DNA repair defects may not be the cause of the observed phenomenon (Figures 1a, c, and g and Supplementary Figures S1a and b). As BRCA1, in contrast to BRCA2, also has an E3 ubiquitin ligase activity and, thus, may require a proteasomal degradation of its target proteins for its proper function, we tested two other E3 ligases involved in HR pathway, RNF8 and BARD1, in the same assay. Not surprisingly, depletion of BARD1, which is essential for the E3 ligase activity of BRCA1, results in a sensitization of HeLa cells to bortezomib, similar to BRCA1 (Figure 1h). However, cells with a knockdown of RNF8, an E3 ligase important for recruitment of BRCA1 to sites of DNA damage, were not more sensitive to bortezomib than control cells (Figure 1h), further supporting the view that HR is not involved directly. To test whether proteasome inhibitors could directly induce DNA damage, HeLa cells were treated with 10–100 nM bortezomib for 8 h, and DNA repair foci were visualized by immunofluorescence staining for phosphorylated histone H2AX (*γ*H2AX), BRCA1, or 53BP1. Unlike *γ*-irradiation (5 Gy) used as a positive control, bortezomib did not lead to any increase in the number of DNA repair nuclear foci containing either *γ*H2AX, BRCA1, or 53BP1 (Figure 2), which is consistent with the published data.^22^ BRCA1-depleted HeLa cells treated with bortezomib accumulate *γ*H2AX in the perinuclear regions instead of the nuclear foci (Figure 2e), which serves as a marker of apoptosis rather than DNA damage.^24^ Therefore, the mechanism underlying sensitivity of BRCA1-depleted cells to bortezomib is most likely associated with other functions of BRCA1.

### BRCA1-associated sensitivity to bortezomib is mediated by the RB1 pathway

**Figure 3:**
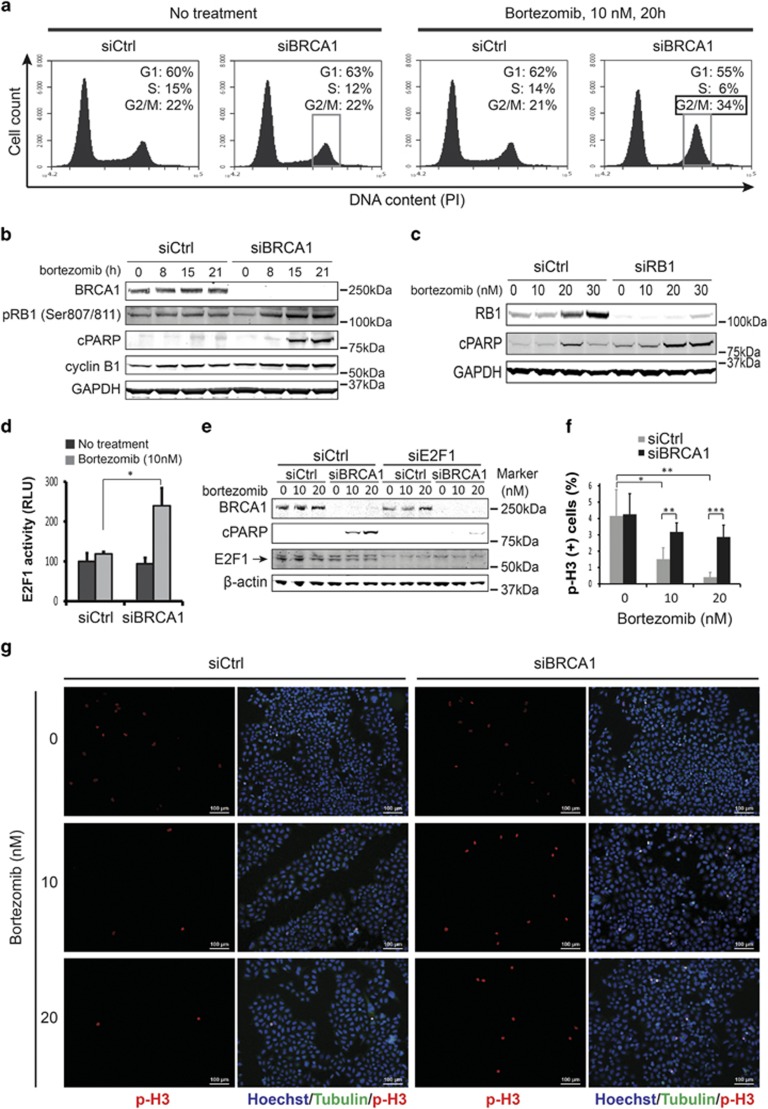
Bortezomib affects both G1/S and G2/M cell cycle checkpoints in BRCA1-depleted cells. (**a**) HeLa cells treated with BRCA1 siRNA (siBRCA1) and 10 nM bortezomib for 20 h are accumulated in the G2 phase of the cell cycle, whereas the number of cells at G1 is reduced. Note that none of these treatments alone had any significant effect on the cell cycle profile. The G2/M fraction is marked with a frame. (**b**) Western blot showing that prolonged treatment of BRCA1-depleted cells with 10 nM bortezomib induced hyperphosphorylation of RB1, accumulation of cyclin B1, and cleaved PARP (cPARP), indicating induction of apoptosis. (**c**) Western blot showing that, similar to BRCA1, depletion of RB1 alone also leads to induction of apoptosis after bortezomib treatment. (**d**) E2F1 reporter activity was measured in HeLa cells transfected with control siBRCA1 and treated with bortezomib. Reporter activity without bortezomib treatment was used as a reference. Error bars indicate S.E.M. from duplicates. (**e**) Western blot demonstrating that knockdown of E2F1 rescues bortezomib-induced apoptosis in BRCA1-depleted cells as judged by a reduction in cPARP level. (**f** and **g**) Immunofluorescence (IF) staining for phosphorylated histone H3 (p-H3) marking mitotic cells reveals that depletion of BRCA1 allows for aberrant entry into mitosis after bortezomib treatment. Example IF images and quantification of p-H3-positive cells are shown in (**g** and **f**), respectively. Error bars represent S.D. with *N*=6 for siControl and *N*=8 for siBRCA1. Statistically significant differences are labeled with asterisks. RLU, relative luciferase unit

Besides DNA repair, BRCA1 regulates the cell cycle in part due to its E3 ubiquitin ligase activity.^25^ We investigated whether a combination of BRCA1 depletion and bortezomib treatment could have a synergistic effect on cell cycle progression distinct from each of the treatments alone. We transfected HeLa cells with BRCA1 or siCtrl, and treated with a minimal effective dose of bortezomib (10 nM) for 20 h before cells were processed for cell cycle analysis by the fluorescence-assisted flow cytometry. We found that the number of BRCA1-depleted cells at the G1 phase noticeably decreased after bortezomib treatment (55%) compared with all other conditions (60–63%), suggesting that the G1/S checkpoint was abrogated (Figure 3a).

This checkpoint is mainly regulated by TP53, RB1, and some other factors.^26^ To address the role of TP53 in BRCA1-mediated toxicity to bortezomib, we knocked down both p53 and BRCA1 in HeLa or U2OS cells, followed by bortezomib treatment. Our results suggest that TP53 does not have any significant role in increased sensitivity to bortezomib after BRCA1 depletion and is probably not responsible for the apparent abrogation of the G1/S checkpoint (Supplementary Figure S2). In contrast, phosphorylation of RB1 at Ser807/811 was noticeably increased after bortezomib treatment in BRCA1-depleted cells but not in control cells transfected with a non-targeting siRNA (Figure 3b). The hyperphosphorylation of RB1 preceded the onset of apoptosis as measured by cleaved PARP (Figure 3b), suggesting that RB1 is likely responsible for abrogation of the G1/S checkpoint and mediates the BRCA1-dependent toxicity.

As hyperphosphorylation of RB1 functionally inactivates the protein,^27^ we hypothesized that an siRNA-mediated depletion of pRB1 should produce the same effect as depletion of BRCA1 when cells were treated with bortezomib. Indeed, knockdown of RB1, but not siCtrl, led to the accumulation of cleaved PARP after bortezomib treatment in a dose-dependent manner (Figure 3c). Phosphorylated RB1 releases E2F family transcription factors, which then induce the expression of multiple target genes mediating transition from G1 into the S phase.^28^ E2F1 is often the most essential factor within the E2F family.^29^ Using an E2F1-specific reporter,^30^ we could demonstrate that the E2F1 activity is indeed increased more than twofold in BRCA1-depleted cells treated with bortezomib, but in none of the treatments alone (Figure 3d). Knockdown of E2F1 greatly diminished the bortezomib-induced apoptosis in BRCA1-depleted cells (Figure 3e), thus providing further evidence for the involvement of the RB1-E2F1 pathway in BRCA1-mediated response to bortezomib.

### Inactivation of 53BP1 rescues BRCA1-mediated sensitivity to bortezomib

We also found that 34% of cells depleted for BRCA1 and treated with bortezomib accumulated at the G2/M phase in contrast to 21–22% for control cells or those treated with bortezomib or siBRCA1 alone (Figure 3a). This is consistent with a steady increase of cyclin B1, a marker of late G2 phase and mitotic prophase, as early as 8 h after bortezomib treatment in BRCA1-depleted cells but not in control cells (Figure 3b). To distinguish between cells in G2 and M phases, we performed an immunofluorescence staining for a mitotic marker histone H3 phosphorylated at Ser10.^31^ As evidenced from Figures 3f and g, the number of mitotic cells in the control decreased from 4% in untreated cells to 1.5% after treatment with 10 nM bortezomib, and further to 0.3% after 20 nM bortezomib. However, the number of p-H3-positive cells within siBRCA1-treated population did not decrease by more than 1% even after treatment with the highest dose of bortezomib, suggesting that the G2/M checkpoint was compromised. Nevertheless, the observed 3% difference in the number of mitotic cells between *BRCA1*-depleted and control samples did not fully account for 12% increase in the G2/M population as shown in Figure 1a. This suggests that inhibition of proteasome activity in *BRCA1*-depleted cells leads to the accumulation of cells at the G2 phase owing to the activation of the G2/M cell cycle checkpoint, which, however, is deficient and allows significant number of cells to prematurely enter mitosis.

**Figure 4:**
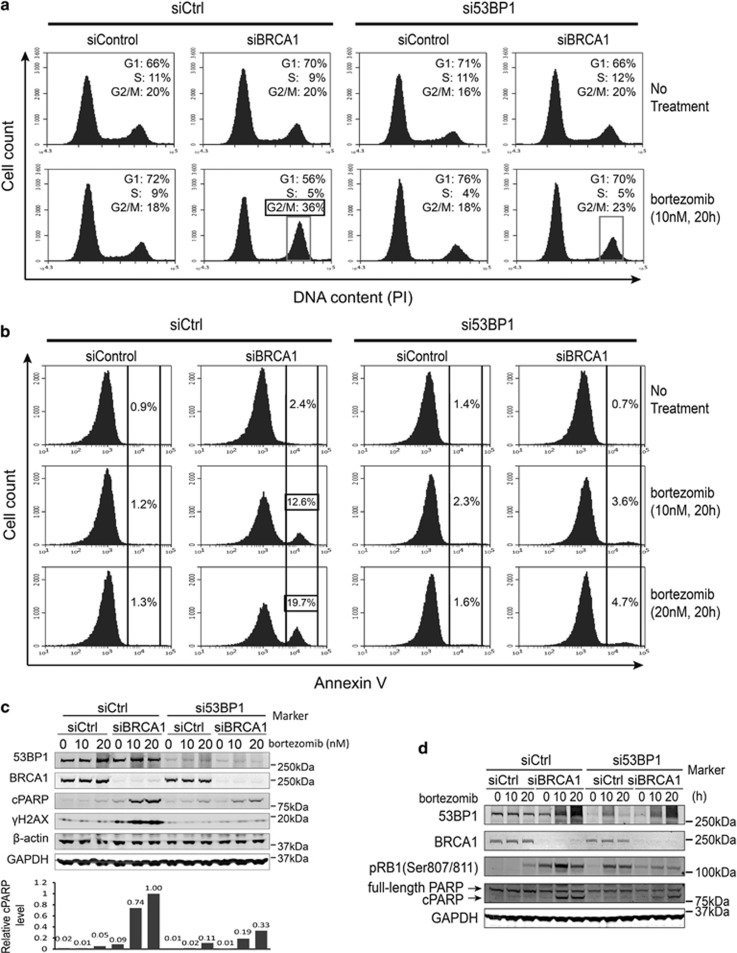
Suppression of 53BP1 inhibits apoptosis in BRCA1-depleted cells after bortezomib treatment. (**a**) FACS analysis of cell cycle distribution of HeLa cells transfected with siRNAs shown above and drug treatment marked on the right. Note that a combination of siBRCA1 and bortezomib treatments leads to a reduced proportion of cells at the G1 phase and increase at the G2/M phase, whereas a concomitant depletion of 53BP1 restores cell cycle distribution to the level of controls. The G2/M fraction is marked with a frame. (**b**) FACS analysis of HeLa cells stained for Annexin V. Annexin V-positive fraction is marked with brackets. Cells were transfected with siRNAs as indicated above and treated with bortezomib as shown on the right. Note that the Annexin V-positive fraction of BRCA1-depleted cells is increasing in a dose-dependent manner after bortezomib treatment, but greatly reduced upon depletion of 53BP1. (**c**) Western blot showing that knockdown of 53BP1 inhibits apoptosis in BRCA1-depleted cells after bortezomib treatment. The bar graph below shows quantification of cleaved PARP (cPARP) level normalized to the average of *β*-actin and glyceraldehyde 3-phosphate dehydrogenase (GAPDH) protein levels. (**d**) Western blot showing inhibition of RB1 phosphorylation in siBRCA1 and bortezomib-treated cells after depletion of 53BP1

Interestingly, inactivation of 53BP1, the best-known factor associated with therapy resistance of BRCA1 mutant cells, was shown to regulate intra-S and G2/M checkpoints.^32^ We wondered whether 53BP1 could have a similar role in the BRCA1-mediated response to proteasome inhibitors. We knocked down both BRCA1 and 53BP1 in HeLa cells, treated them with 10 nM bortezomib for 20 h, and performed a cell cycle profiling as before (Figure 4a). Indeed, depletion of 53BP1 decreased the G2/M peak almost to the level of control cells in siBRCA1/bortezomib-treated cells (Figure 4a) supporting the role of 53BP1 at the G2/M checkpoint. At the same time, the G1 peak was restored to the level of control samples (Figure 4a), suggesting that 53BP1 is the most proximal regulatory factor of BRCA1 acting upstream of the RB1-E2F pathway in this context. Accordingly, depletion of 53BP1 resulted in reduced levels of apoptosis as measured by Annexin V-mediated detection of phosphatidylserine on the cell surface and the level of cleaved PARP (Figures 4b and c), and phosphorylated RB1 protein (Figure 4d).

### Additional factors affect BRCA1-mediated response to bortezomib

**Figure 5:**
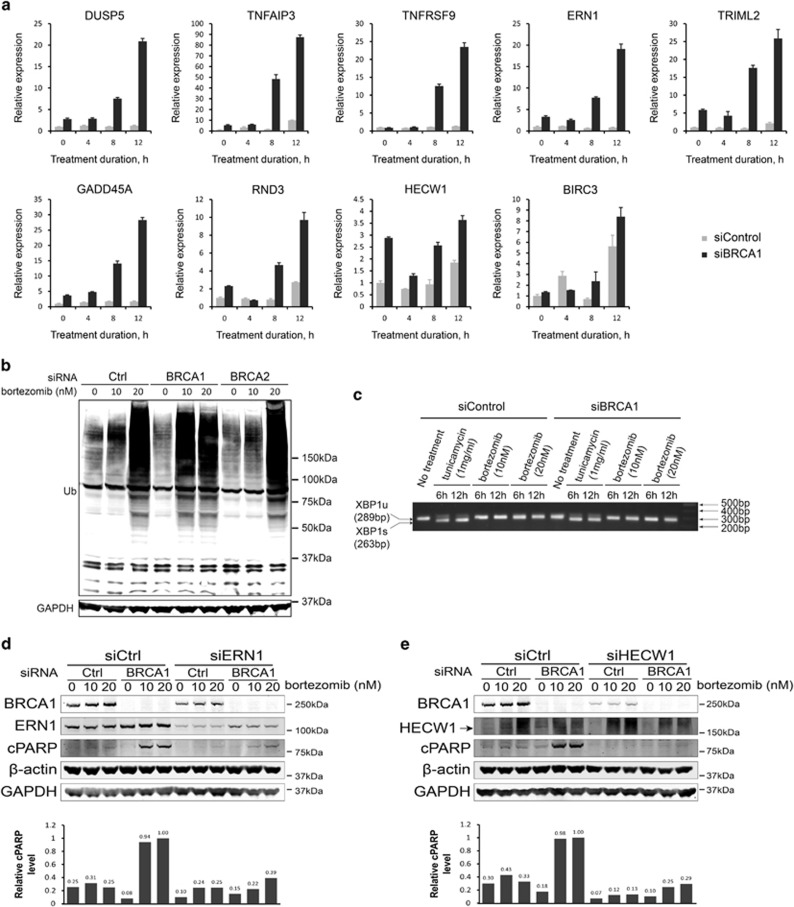
Additional factors are critically involved in bortezomib-induced apoptosis of BRCA1-depleted cells. (**a**) qRT-PCR validation of differentially expressed genes initially identified by a microarray analysis in the course of bortezomib treatment (10 nM) with or without BRCA1 depletion. (**b**) Western blot showing a bulk of ubiquitinated proteins accumulated after exposure to 10 nM bortezomib in BRCA1- but not BRCA2-depleted HeLa cells. (**c**) PCR analysis showing lack of XBP1 splicing after bortezomib treatment. Tunicamycin treatment is used as a positive control. (**d**) Western blot demonstrating that knockdown of ERN1 results in a substantial reduction in the amount of cleaved PARP (cPARP) protein in BRCA1-depleted cells treated with bortezomib. The bar graph below shows quantification of cPARP level normalized to the average of *β*-actin and glyceraldehyde 3-phosphate dehydrogenase (GAPDH) protein levels. (**e**) Same as (**d**) for HECW1

To obtain a comprehensive view of the BRCA1-mediated cellular response to bortezomib, we performed a gene expression microarray analysis of HeLa and U2OS cells transfected with siBRCA1 or siControl with or without bortezomib treatment. After reannotation and normalization, all non-protein-coding genes except miRNAs were excluded from further analysis. We were interested in such genes whose expression ratio before and after bortezomib treatment in BRCA1-depleted cells exceeded an analogous ratio in siCtrl-transfected cells by more than twofold. In addition, the amplitude and direction of the gene expression changes should be consistent between HeLa and U2OS cells. Such genes were considered functionally significant candidates (Supplementary Figure S3). Expression of nine genes related to control of cell growth and apoptosis was verified by quantitative reverse transcription-PCR (qRT-PCR) (Figure 5a). Interestingly, most of them were to a various degree upregulated in BRCA1-knockdown cells even without bortezomib treatment, and their expression further increased rather sharply 8- to 50-fold after 8 h of bortezomib treatment just about 2 h before the onset of apoptosis (Figure 5a).

To investigate a functional significance of these differentially expressed genes, we tested two to four independent siRNAs for each gene for the ability to prevent apoptosis of siBRCA1-depleted HeLa cells after bortezomib treatment (Supplementary Figure S5). We found that siRNAs targeting ERN1, HECW1, DUSP5, TRIML2, and BIRC3, but not DAPK2, could rescue the cells from apoptosis (Supplementary Figure S5). Unfortunately, specificity of the DUSP5, TRIML2, and BIRC3 knockdowns could not be unequivocally confirmed at the protein level, and the role of these genes was not investigated any further. However, an early induction of ERN1, a major regulator of the unfolded protein response (UPR) also known as IRE1, appeared particularly relevant in connection with a known E3 ubiquitin ligase activity of BRCA1 and potentially impaired degradation of ubiquitinated proteins due to proteasome inhibition with bortezomib.^33^ Accumulation of large amounts of ubiquitinated proteins in HeLa cells treated with 10 nM bortezomib became evident only after BRCA1 depletion, whereas control or BRCA2-depleted cells required two times as much bortezomib to produce a similar effect (Figure 5b). This suggested that inhibition of BRCA1 directly or indirectly leads to a massive protein destabilization requiring proteasome activity or induction of UPR. In a canonical pathway, ERN1 is an endoribonuclease that, upon activation, catalyzes splicing of the X-boxbinding protein 1 (XBP1) mRNA, inducing transcription of chaperones and triggering growth arrest and apoptosis.^34^ Surprisingly, despite a strong transcriptional activation of ERN1, we could not detect any splicing events of XBP1 mRNA in BRCA1-depleted cells treated with bortezomib (Figure 5c). Alternatively, ERN1 could trigger a so-called RIDD (regulated IRE1-dependent decay) pathway of UPR, in which ERN1 induced degradation of a subset of mRNAs independently of XBP1.^35^ However, there are no clear candidate target genes to test this hypothesis. One way or another, knockdown of ERN1 significantly inhibited induction of apoptosis, suggesting that it has an essential role in the process (Figure 5d).

Knockdown of another siBRCA1/bortezomib-induced gene *HECW1* completely rescued HeLa cells from apoptosis (Figure 5e). Three out of four siRNAs (all except siHECW1–5) that were tested produced a similar effect, implying a critical role for this gene in BRCA1-mediated sensitivity to bortezomib (Supplementary Figure S5k). HECW1 is an HECT, C2, and WW domain containing E3 ubiquitin protein ligase also known as NEDL1.^36^ No data are available regarding its possible interaction with BRCA1 or ERN1. However, the protein level of ERN1 is not affected by the knockdown of HECW1 and *vice versa*, suggesting their independence (data not shown).

Finally, three other differentially expressed genes *TNFAIP3*, *TNFRSF9*, and *TNFAIP8L1* regulate the tumor necrosis factor (TNF) signaling and cytokine response, and could therefore be involved in the execution of bortezomib-induced cell death via TNF-related receptors.^37^ Indeed, protein profiling using an apoptosis protein array revealed a strong and specific induction of the death receptor 4 (DR4/TRAILR1) (Supplementary Figure S4). A knockdown of TNFRSF9 or DR4 effectively suppressed the induction of apoptosis in BRCA1-depleted bortezomib-treated HeLa cells (Supplementary Figures S4e and c, respectively). Taken together, our data demonstrate that cellular toxicity to bortezomib associated with a loss of BRCA1 involves RB1-mediated G1/S checkpoint inactivation, 53BP1-mediated G2/M checkpoint activation, a cascade of deregulated E3 ubiquitin ligases, and UPR, culminating in a TRAILR1-mediated apoptosis.

## Discussion

This is the first report suggesting that BRCA1 has a 26S proteasome-related activity critical for cell viability. BRCA1 is a multifunctional protein serving as a protein–protein interaction platform, a phosphorylation substrate for various kinases, and an E3 ubiquitin protein ligase. As the 26S proteasome degrades ubiquitinated proteins, the E3 ubiquitin ligase activity of BRCA1 is most likely responsible for the response to bortezomib. Our finding that depletion of BARD1, a RING domain protein required for the E3 ubiquitin ligase activity of BRCA1, but not other HR proteins with or without an E3 activity, produces a similar phenotype in response to proteasome inhibition further supports this idea. Several ubiquitination targets of BRCA1 have been identified. It was demonstrated that BRCA1 regulates the G2/M cell cycle checkpoint by ubiquitinating cyclin B and Cdc25C.^12^ This is consistent with our observation that BRCA1-depleted cells accumulate in mitosis becoming positive for phosphorylated histone H3, which, however, does not occur without inhibition of the proteasome function. A premature entry into mitosis is associated with cell death owing to the so-called mitotic catastrophe,^38^ which is probably the reason for observed reduction in survival of BRCA1-depleted cells after bortezomib treatment. On the other hand, sensitivity of BRCA1-depleted cells to bortezomib may be associated with the accumulation of large amounts of potentially toxic ubiquitinated proteins, which suggests that BRCA1 could have a role in a global protein stability.

In addition, the proteasome-related activity of BRCA1 appears to be tightly linked to the RB1-mediated cell cycle checkpoints. This is consistent with previous reports showing that BRCA1-induced growth arrest is RB1-dependent, and BRCA1 physically binds a hypophosphorylated RB1 protein.^39^ We show that RB1 is strongly hyperphosphorylated in BRCA1-depleted cells after bortezomib treatment, suggesting that BRCA1 may protect RB1 from phosphorylation. Phosphorylation of RB1 protein is mediated by cyclin D/Cdk4/6 and cyclin C/Cdk3 complexes.^28, 40, 41^ BRCA1 binds cyclin D family proteins and Cdk4 via its RING domain.^42^ Available literature is contradictory, arguing that BRCA1 may stimulate or inhibit the expression of cyclin D1 under different circumstances.^43, 44^ Nevertheless, the importance of the RB1-E2F pathway for the proteasome-dependent activity of BRCA1 is testified by the fact that knockdown of RB1 phenocopies the toxic effect of BRCA1 depletion in response to bortezomib, and the fact that this toxicity can be essentially blocked by depletion of E2F1.

Our data also reveal a critical role of 53BP1 in regulating BRCA1-mediated response to proteasome inhibition. 53BP1 is primarily known as an antagonist of BRCA1 during repair of double-stranded DNA breaks. 53BP1 protects broken DNA ends from extensive resection by exonucleases, such as CtIP, thus promoting DNA repair via the non-homologous end-joining mechanism rather than HR mediated by BRCA1. However, we did not find any evidence that bortezomib treatment could directly induce excessive DNA damage, which could explain the requirement for BRCA1 function. Instead, expression of 53BP1 is critical for activating the G2/M cell cycle checkpoint in BRCA1-deficient cells after bortezomib treatment. A role in G2/M checkpoint has been reported for 53BP1, although only following DNA damage.^45^ Therefore, the proteasome-related activity of BRCA1 may indirectly involve its DNA repair-associated function.

Taken together, we found that BRCA1 is required for cell survival when their proteasome function is inhibited, and identified several genes, including *53BP1*, *RB1*, *E2F1*, *HECW1*, and *ERN1*, whose expression can modulate this cytotoxic response.

## Materials and Methods

### Cell culture

Cell lines MDA-MB-231, HeLa, and U2OS were obtained from the American Type Culture Collection (LGC Standards GmbH, Wesel, Germany). The cell line authenticity was verified at the FIMM technology center using a StemElite ID System (Promega, Madison, WI, USA). Cells were grown in Dulbecco's modified Eagle's medium (DMEM) supplemented with 10% fetal bovine serum, L-glutamine (2 mM) and 1 × penicillin–streptomycin (Life Technologies, Thermo Fisher Scientific, Waltham, MA USA) at 37 °C in a 5% CO_2_ atmosphere. *R26*^CreERT2/RMCE^*; Brca1*^SCo/Δ^ ES cells provided with a human *BRCA1* cDNA or empty RMCE vector were cultured as described.^21^ Mouse *Brca1* was deleted by incubating overnight with 0.5 *μ*M 4-OHT (Sigma, St. Louis, MO, USA). One week later, cells were seeded in triplicate at 1000 cells per well in 96-well plates for drug sensitivity testing as described.^46^ Brca1-deleted cells with an empty RMCE vector were seeded at 1250 cells per well to compensate for their proliferation defect.

### Gene silencing

siRNAs were purchased from Qiagen (Valencia, CA, USA). siRNA names, catalog numbers, and target sequences are listed in Supplementary Table S1. For drug sensitivity testing, 80–90% confluent cells seeded in 6-cm dishes one day before were transfected with siRNA duplexes at 20 nM final concentration using Lipofectamine RNAiMAX (Invitrogen, Carlsbad, CA, USA) according to the manufacturer's instructions. For all other applications, except microarray analysis, cells were transfected with siRNA duplexes at a final concentration 10 nM and used 24 h after transfection.

### High-throughput chemical compound screening

The screening was performed essentially as described by Pemovska *et al.*^19^ Compounds were dissolved in corresponding media as described and automatically dispensed into 384-well plates containing 5 *μ*l in each well. Cells transfected with siBRCA1 or siCtrl one day before were trypsinized and added to the drug-containing plates at 4000 cells per well. After 72 h incubation at 37 °C and 5% CO_2_, cell viability was measured using CellTiter-Blue fluorescent assay (Promega) as described below. Dose–response curves were generated using the Studies software (Dotmatics Ltd, Bishop's Stortford, Herts, UK) as described earlier.^19^ Drug sensitivity scores were calculated for each drug to quantitatively profile the samples.^19, 47^ Briefly, the logistic curve-fitting parameters were used to calculate the area under the dose–response curve, relative to the total area between 10% threshold and 100% inhibition, which was further normalized by a logarithm of the top response.

### Drug sensitivity testing

Twenty-four hours after siRNA transfection, cells were resuspended and seeded into 96-well plates at 11 000 cells per well (MDA-MB-231), 4000 cells per well (HeLa), or 5000 cells per well (U2OS), and 100 *μ*l per well. The remaining cells were pelleted for western blot to determine the knockdown efficiency for each siRNA. Serial dilutions of bortezomib (Selleck Chemicals, Houston, TX, USA) and carfilzomib (ChemieTek, Indianapolis, IN, USA) were prepared in DMEM and added to cells in a 50 *μ*l volume. After 72 h, CellTiter-Blue (Promega) was added to each well at a final concentration of 10%, incubated for 3 h, and fluorescence was measured at a wavelength of 520 nm using a PHERAStar FS plate reader (BMG Labtech). Cell viability curves were plotted using the GraphPad Prism software (GraphPad, La Jolla, CA, USA).

### Western blotting

Cells were prepared with a standard procedure using a modified RIPA buffer (50 mM Tris-HCl (pH 8.0), 250 mM NaCl, 2 mM EDTA, 1.0% Triton X-100, 0.5% sodium deoxycholate, 0.1% SDS, 5 mM NaF and 5 mM Na_3_VO_4_) supplemented with a protease inhibitor tablet (Pierce, Waltham, MA, USA). Protein concentrations were determined using the BCA Protein Assay Reagent (Pierce). Protein electrophoresis was carried out using NuPage 4–12% Bis-Tris precast gels (Invitrogen) according to the manufacturer's instructions, followed by blotting to a nitrocellulose membrane (Millipore, Billerica, MA, USA). Membranes were incubated overnight at +4 °C in a blocking buffer (1xTBS, 0.05% Tween-20, 5% milk) with the following primary antibodies: BRCA1 (OP92) and BRCA2 (OP95) (Calbiochem, Billerica, MA, USA); 53BP1 (ab21083) and phospho-H2AX (Ser139; ab22551) (Abcam, Cambridge, UK); p53 (DO-5; sc-126), ubiquitin (P4D1; sc-8017), cyclin B1 (H-433; sc-752), and E2F1 (C-20; sc-193) (Santa Cruz Biotechnologies, Dallas, TX, USA); cleaved PARP (Asp214; no. 9541), RB1 (4H1; no. 9309), phospho-RB1 (Ser807/811; no. 9308), phospho-histone H3 (Ser10; no. 3377) (Cell Signaling Technology, Danvers, MA, USA); GAPDH (NB300-285) and *β*-actin (NB600-505) (Novus Biologicals, Littleton, CO, USA); and p21 (556430; BD Biosciences, San Jose, CA, USA). After incubation with fluorescently labeled secondary antibodies (goat-anti-mouse IRDye 800 CW or goat-anti-rabbit IRDye 680 LT; LI-COR Biosciences, Lincoln, NE, USA) diluted 1 : 10 000, membranes were scanned using a fluorescence scanner Odyssey (LI-COR Biosciences).

### Immunofluorescence

To visualize DNA repair complexes, cells grown on glass coverslips for 36 h were *γ*-irradiated (10 Gy) or treated with bortezomib (10 nM). After 8 h, cells were fixed with 2% paraformaldehyde diluted in PBS containing 1 mM CaCl_2_ and 0.5 mM MgCl_2_ (PBS^++^) for 15 min. After three washes with PBS^++^, cells were permeabilized with 0.5% Triton X-100 for 15 min. Then, they were blocked for 30 min in an incubation buffer (0.5% BSA, 0.15% glycine, 0.1% Triton X-100 in 1 × PBS). After blocking, the cells were incubated overnight at 4 °C with mouse-anti-phospho-H2AX (Ser139, ab22551; Abcam) and rabbit-anti-BRCA1 (C-20, sc-642; Santa Cruz Biotechnologies) or rabbit-anti-53BP1 (ab21083; Abcam) antibodies. Secondary goat-anti-mouse Alexa 488 and goat-anti-rabbit Alexa 594 antibodies were applied for 1 h at room temperature after three washes with the incubation buffer. Images were taken using Nikon Eclipse 90i (Tokyo, Japan) fluorescent microscope and processed with the Nikon NIS-Elements AR software (Tokyo, Japan).

### Cell cycle analysis

Cell cycle distribution was analyzed based on DNA staining using the Propidium Iodide (PI) Staining Solution (00-6990; eBioscience, San Diego, CA, USA) according to the manufacturer's protocol. Briefly, cells seeded in six-well plates were transfected with siRNAs and treated with bortezomib for 20 h. Cells were then washed with cold PBS, and fixed with cold 70% ethanol overnight. The PI staining was carried out by resuspending the cells in 500 *μ*l PI/Triton X-100 solution (0.1% Triton X-100 in PBS, 0.2 mg/ml DNAse-free RNAse A, and PI Staining Solution diluted 1 : 200) for 30 min at room temperature. Cell cycle data were acquired using a BD Accuri C6 Flow Cytometer Instrument (BD Biosciences) and analyzed with the CFlow Sampler software (BD Biosciences).

### Gene expression microarray analysis

HeLa and U2OS cells grown in 6 cm dishes were transfected with siBRCA1 or siCtrl at 20 nM final concentration. After 24 h, cells were treated with 20 nM bortezomib for 8 h, after which a total RNA was extracted with a Nucleospin RNA II Kit (Macherey-Nagel, Düren, Germany). RNA quality was tested using Agilent 2100 Bioanalyzer instrument (Agilent Technologies, Santa Clara, CA, USA) and hybridized using an Affymetrix GeneChip Human Gene 2.0 ST Array at the Biomedicum Helsinki FuGu facility, University of Helsinki (Helsinki, Finland). Raw data files (.CEL files) were imported into R v. 2.13 software (http://cran.r-project.org) and analyzed with the Bioconductor software suite (http://www.bioconductor.org). Briefly, after quality check, the data were reannotated according to the Ensembl gene and transcript databases with the brainarray custom cdf v.16 application (http://brainarray.mbni.med.umich.edu/Brainarray/Database/CustomCDF/16.0.0/ensg.asp) and preprocessed by the RMA algorithm.^48^ The microarray results are publicly available in the GEO database (ID GSE56280).

### Quantitative reverse transcription-PCR

Cells grown in 24-well plates were transfected with siRNAs, and treated with bortezomib for an indicated period 24 h later. A total RNA was extracted with a Nucleospin RNA II Kit (Macherey-Nagel) and 1 *μ*g RNA was used as a template for cDNA synthesis using a RevertAid First Strand cDNA Synthesis Kit (Thermo Scientific, Waltham, MA, USA). Real-Time qPCR was carried out using the SYBR Green detection method and a Bio-Rad C1000 cycler (Bio-Rad Laboratories, Hercules, CA, USA). Data were analyzed with the CFX Manager software (Bio-Rad Laboratories). All primer sequences are shown in Supplementary Table S3.

### Statistical analysis

All experiments were repeated at least two times. Error bars represent S.D. calculated from two to eight technical replicas for each data point for plate-based drug sensitivity testing, or from three technical replicas for qRT-PCR experiments. Statistical significance was evaluated using a two-tailed Student's *t*-test with unequal variance. Statistically significant differences are shown as ***P<0.001, **P<0.01, and *P<0.05.

## Abbreviations

BRCT (domain): BRCA1 C terminus
DSBs: double-stranded DNA breaks
FDA: US Food and Drug Administration
HR: homologous recombination
PARP: poly-(ADP-ribose) polymerase
PBS: phosphate buffered saline
RING (domain): really interesting new gene
siRNA: small interfering RNA
TNF: tumor necrosis factor
UPR: unfolded protein response
