# Supplementary figures and images for: Suppression of BRCA1 sensitizes cells to proteasome inhibitors
Source: Cell Death Dis. 2014 Dec 18;5(12):e1580–. doi: 10.1038/cddis.2014.537 (PMC4649846; doi:10.1038/cddis.2014.537)

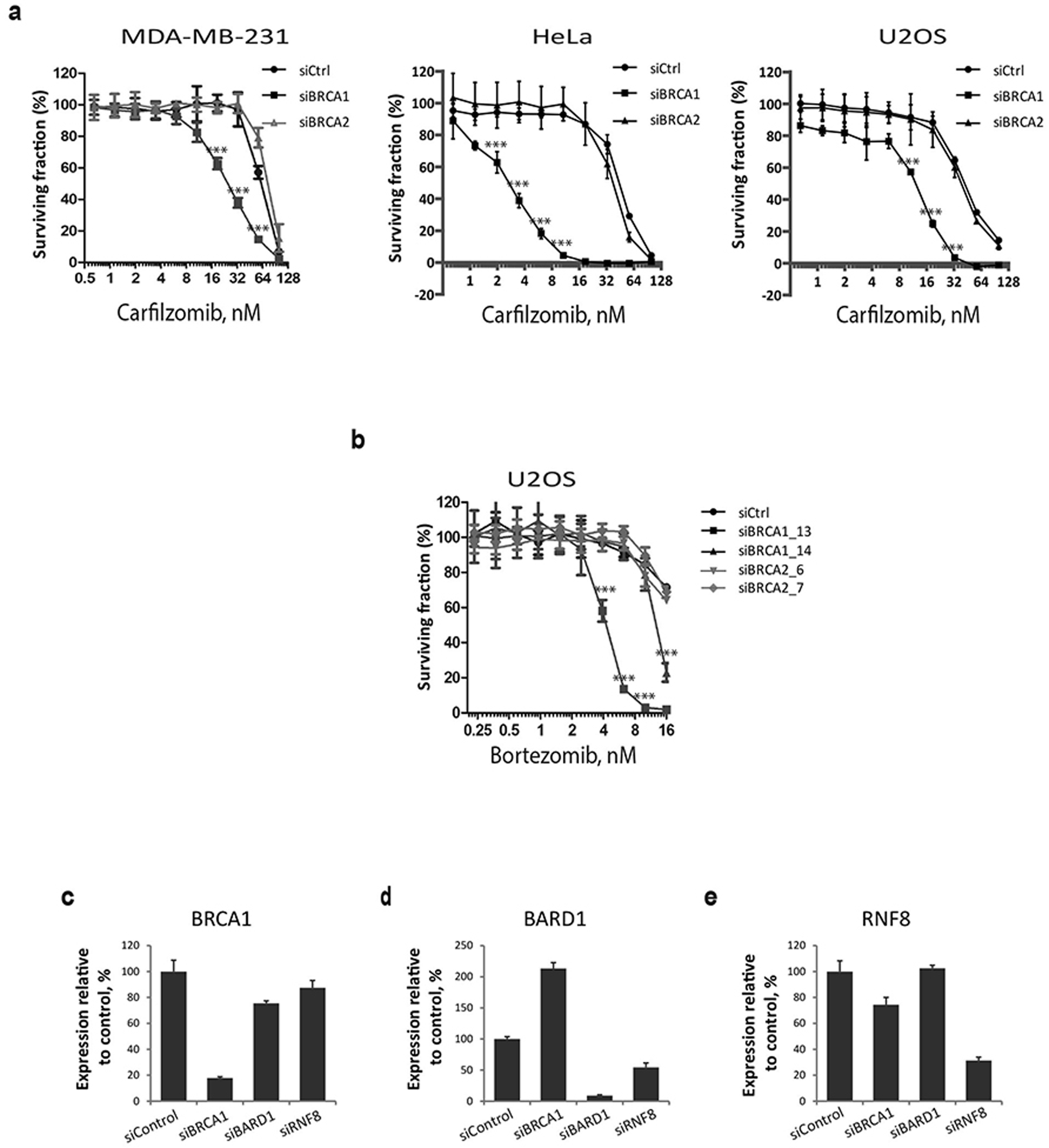

Supplement: Supplementary Figure 1 [file cddis2014537x1.tif]

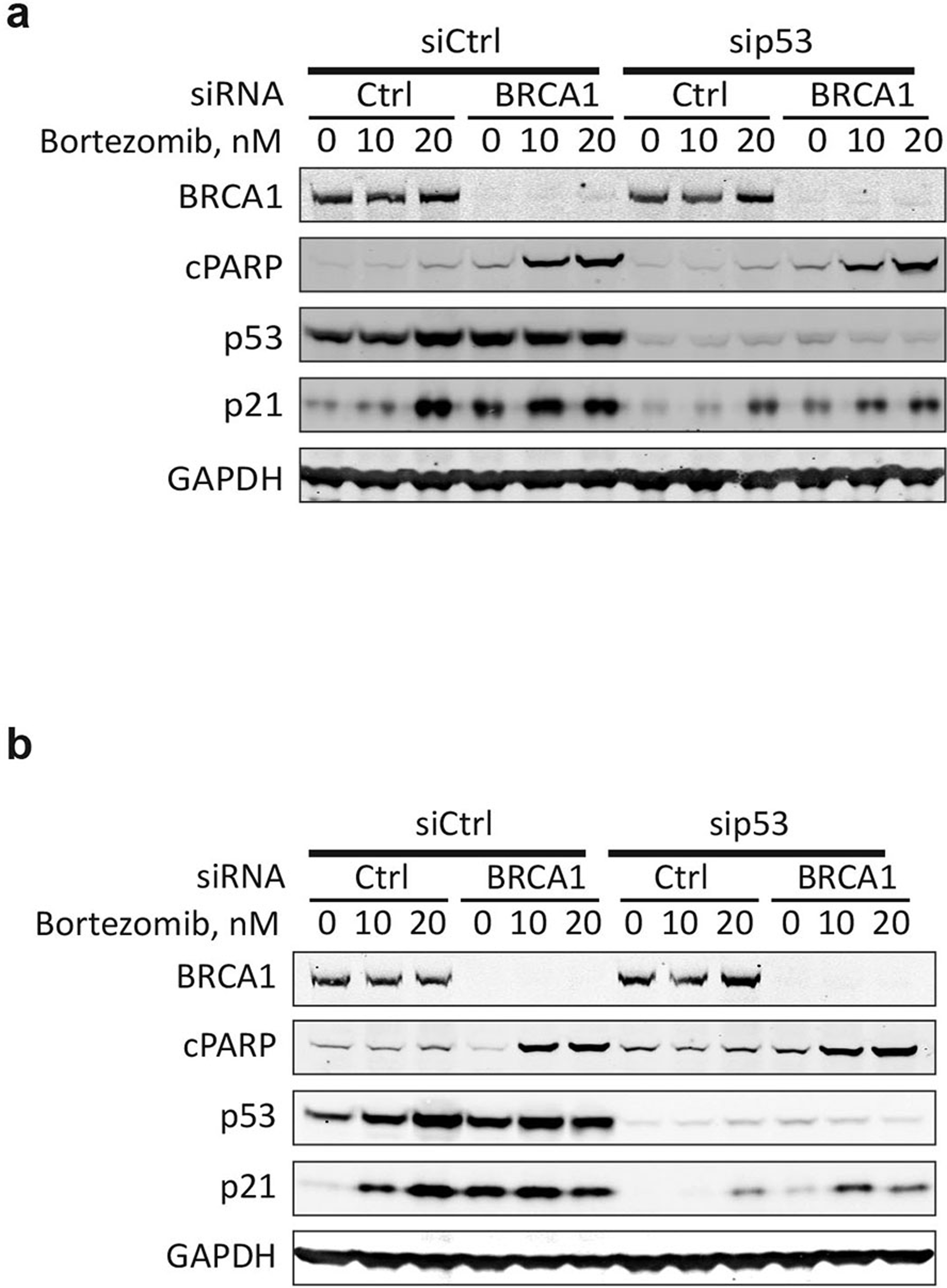

Supplement: Supplementary Figure 2 [file cddis2014537x2.tif]

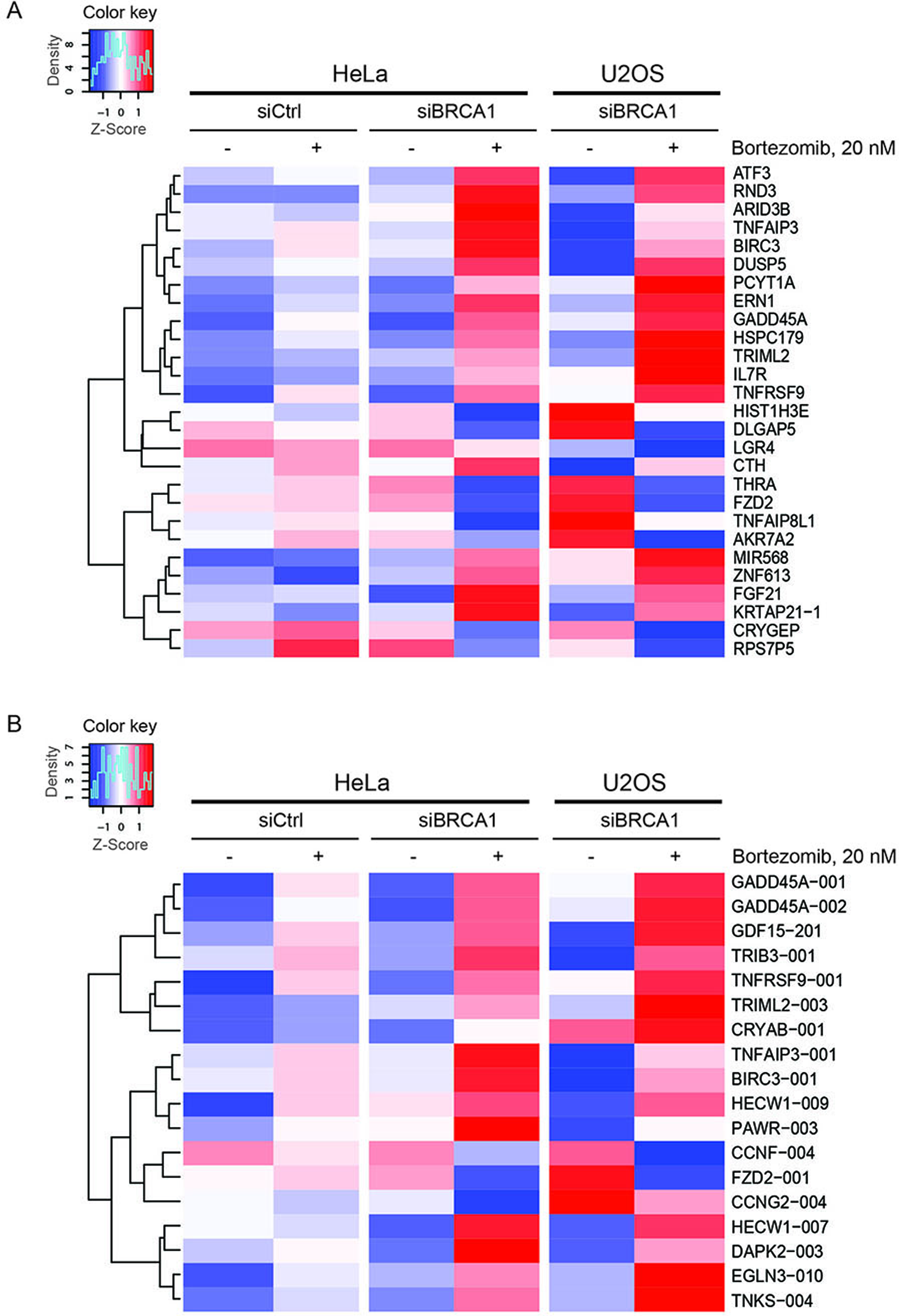

Supplement: Supplementary Figure 3 [file cddis2014537x3.tif]

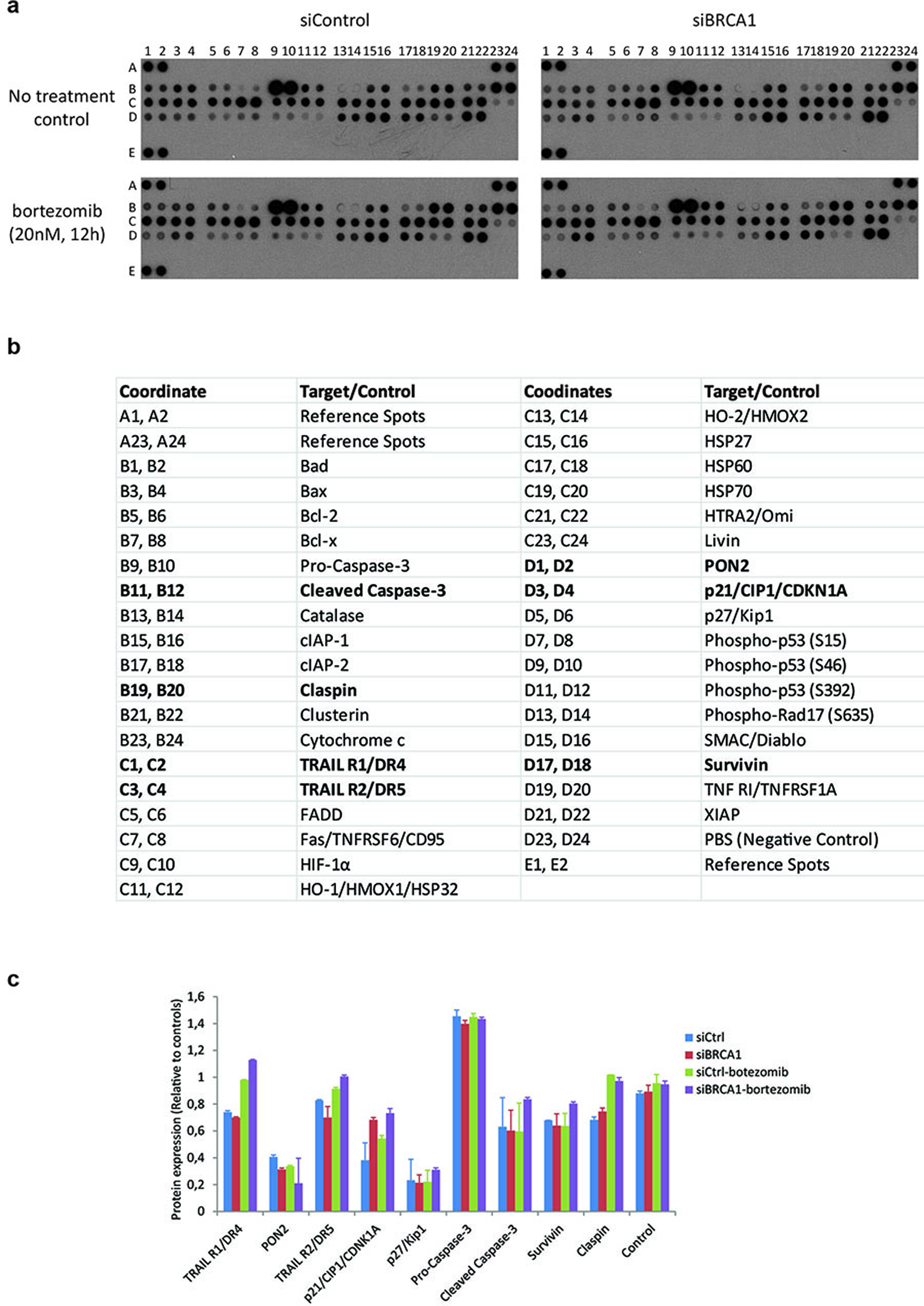

Supplement: Supplementary Figure 4 [file cddis2014537x4.tif]

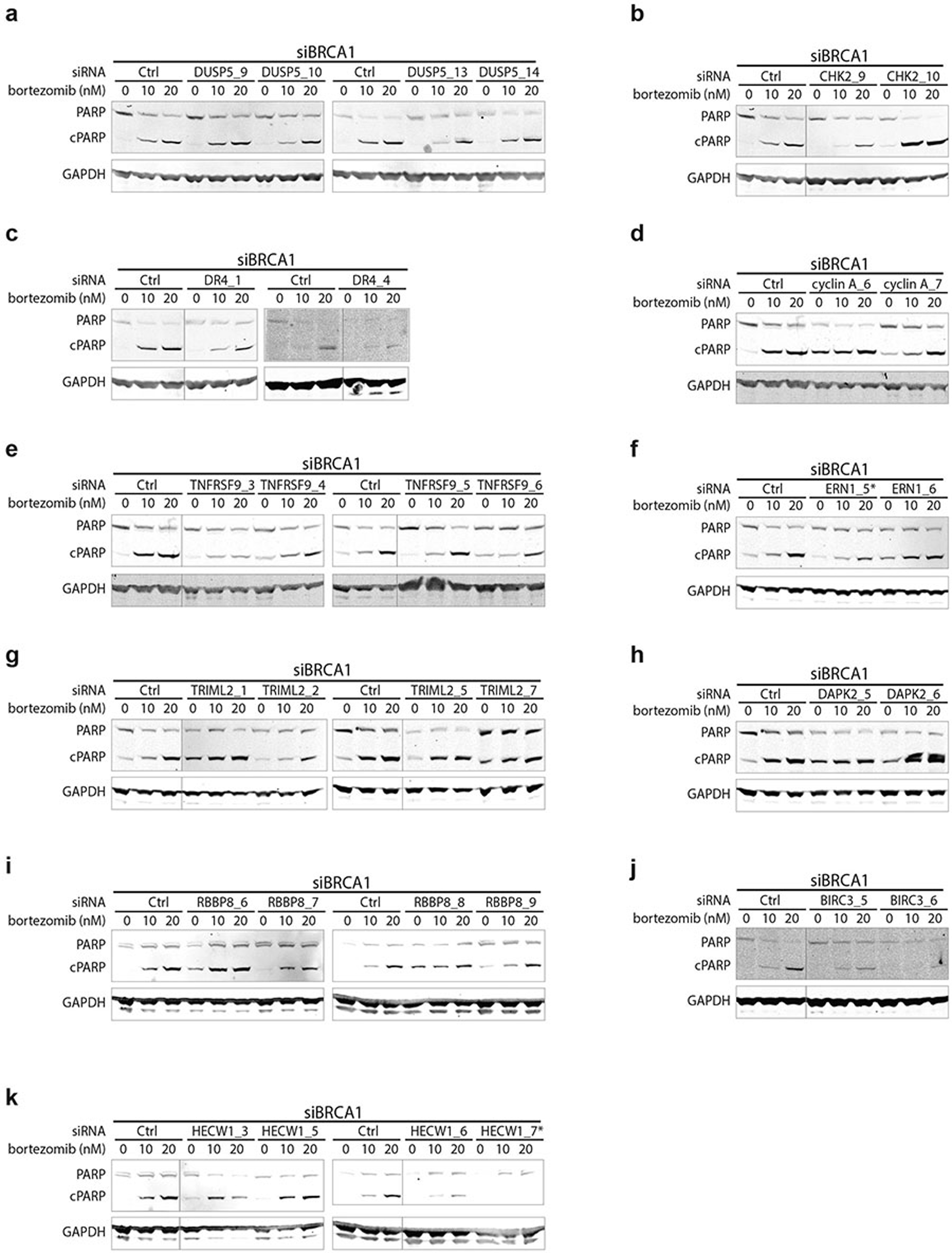

Supplement: Supplementary Figure 5 [file cddis2014537x5.tif]
