# Supplementary material for: Suppression of BRCA1 sensitizes cells to proteasome inhibitors
Source: Cell Death Dis. 2014 Dec 18;5(12):e1580–. doi: 10.1038/cddis.2014.537 (PMC4649846; doi:10.1038/cddis.2014.537)
Supplement: Supplementary Table S1 and S3 [file cddis2014537x6.doc]

**Supplementary Table 1:** siRNAs and their target sequences.

| Target gene | siRNA ID* | Target sequence |
| --- | --- | --- |
| Non-targeting control | Ctrl_AllStars_1 | Not disclosed |
| BRCA1 | Hs_BRCA1_13** | CAGCAGTTTATTACTCACTAA |
| BRCA1 | Hs_BRCA1_14 | CAGGAAATGGCTGAACTAGAA |
| BRCA2 | Hs_BRCA2_6 | CAGGACACAATTACAACTAAA |
| BRCA2 | Hs_BRCA2_7** | TTGGAGGAATATCGTAGGTAA |
| RB1 | Hs_RB1_7** | CGCGTGTAAATTCTAGTGCAA |
| RB1 | Hs_RB1_8 | CAGGGTTGTGTCGAAATTGGA |
| TP53BP1 | Hs_TP53BP1_7** | TTGTTTGACCAGAGCAATAAA |
| TP53BP1 | Hs_TP53BP1_8 | CAGGACAGTCTTTCCACGAAT |
| E2F1 | Hs_E2F1_5** | AACTCCTCGCAGATCGTCATC |
| E2F1 | Hs_E2F1_6 | ACGCTATGAGACCTCACTGAA |
| ERN1 | Hs_ERN1_5** | CAGCACGGACGTCAAGTTTGA |
| ERN1 | Hs_ERN1_6 | CAGGACGTGAGCGACAGAATA |
| HECW1 | Hs_HECW1_7** | AACGGTCGTCTTCCCAATCAT |
| HECW1 | Hs_HECW1_3 | CACCGCGGAAATCGACCTAAA |
| HECW1 | Hs_HECW1_5 | TGCGGTGGAGCGCTTCAATAA |
| HECW1 | Hs_HECW1_6 | CCGGGACTTGGTGAATTTCAT |
| DUSP5 | Hs_DUSP5_9 | CGCGACCCACCTACACTACAA |
| DUSP5 | Hs_DUSP5_10 | CTGACTGTTGCGTGGATGTAA |
| DUSP5 | Hs_DUSP5_13 | CTGCATGGCTTACCTTATGAA |
| DUSP5 | Hs_DUSP5_14 | CAAGTGCGAGTTCCTCGCCAA |
| TNFRSF9 | Hs_TNFRSF9_3 | CAAGAACACCATCCTACATAA |
| TNFRSF9 | Hs_TNFRSF9_4 | CTGGTACATTCTGTGATAATA |
| TNFRSF9 | Hs_TNFRSF9_5 | CTGCCGATTTCCAGAAGAAGA |
| TNFRSF9 | Hs_TNFRSF9_6 | TGGGACATTTAACGATCAGAA |
| TRIML2 | Hs_FLJ25801_1 | CGGGCAGATATCATTCTACAA |
| TRIML2 | Hs_FLJ25801_2 | CAGCATGTTCAGAGTACTCCA |
| TRIML2 | Hs_FLJ25801_5 | CAGGTGGCAAGTGGGCATATA |
| TRIML2 | Hs_FLJ25801_7 | TTGAGTCTGAGTATAGTATGA |
| DR4 | Hs_TNFRSF10A_1 | CAGGAACTTTCCGGAATGACA |
| DR4 | Hs_TNFRSF10A_4 | CAGGCAATGGACATAATATAT |
| BIRC3 | Hs_BIRC3_5 | AATTGGGAACCGAAGGATAAT |
| BIRC3 | Hs_BIRC3_6 | AAGACACTTCAAGATACACAG |
| TNFAIP3 | Hs_TNFAIP3_1 | CCGAGCTGTTCCACTTGTTAA |
| TNFAIP3 | Hs_TNFAIP3_3 | CAGATGTATGGCTAACCGGAA |
| TNFAIP3 | Hs_TNFAIP3_4 | CTCGGCTATGACAGCCATCAT |
| TNFAIP3 | Hs_TNFAIP3_5 | CAGCCTTTACTCATACTATTA |
| CtIP | Hs_RBBP8_6 | CTGGCGTTAACCGGCTACGAA |
| CtIP | Hs_RBBP8_7 | CATGTCCGATACATAGAACAA |
| CtIP | Hs_RBBP8_8 | GACGTCAGCCTTACAACGCAA |
| CtIP | Hs_RBBP8_9 | AAAGACGACTTGATACCTCTA |
| CHEK2 | Hs_CHEK2_9 | ACGCCGTCCTTTGAATAACAA |
| CHEK2 | Hs_CHEK2_10 | AGGACTGTCTTATAAAGATTA |
| Cyclin A2 | Hs_CCNA2_6 | ACCAGAGACACTAAATCTGTA |
| Cyclin A2 | Hs_CCNA2_7 | GCCAGCTGTCAGGATAATAAA |
| DAPK2 | Hs_DAPK2_5 | CGGAATTTGTTGCTCCAGAAA |
| DAPK2 | Hs_DAPK2_6 | CTGGTTAAAGAGACCCGGAAA |
| RNF8 | Hs_RNF8_7 | CCTCATCGTATCTAAGGATAA |
| BARD1 | Hs_BARD1_5 | AACTGGATGTCCAGTGTGTTA |

* siRNA IDs are given according to Qiagen.

**siRNA used when no specific number is indicated

**Supplementary Table 3:** PCR primer sequences.

| Target gene | Primer ID | Primer sequence |
| --- | --- | --- |
| BRCA1 | BRCA1_F | ACAAATACTCATGCCAGCTCAT |
| BRCA1 | BRCA1_R | GGCTCCTTGCTAAGCCAGG |
| BRCA2 | BRCA2_F | AGATAAGTCAGTGGTATGTGG |
| BRCA2 | BRCA2_R | ACTTGACCAAGACATATCAGG |
| Beta-actin | Actin_F | CCCAGCACAATGAAGATCAA |
| Beta-actin | Actin_R | ACTCCTGCTTGCTGATCCAC |
| GAPDH | GAPDH_F | TCTGACTTCAACAGCGACAC |
| GAPDH | GAPDH_R | CCCTGTTGCTGTAGCCAAAT |
| DUSP5 | DUSP5_F | ATGGATCCCTGTGGAAGACA |
| DUSP5 | DUSP5_R | GGAGATCCCAGCCTCACAGT |
| TNFAIP3 | TNFAIP3_F | TGGGACTCCAGAAAACAAGG |
| TNFAIP3 | TNFAIP3_R | ATGGTGTTCTGGAACCTGGA |
| TNFRSF9 | TNFRSF9_F | ACGCTCCGTTTCTCTGTTGT |
| TNFRSF9 | TNFRSF9_R | CTTCTGGAAATCGGCAGCTA |
| ERN1 | ERN1_F | GTGCTCAAACACCCGTTCTT |
| ERN1 | ERN1_R | CTCCCGCCTCTCTCTAACTG |
| TRIML2 | TRIML2_F | GCAAGTCACTGCTGCTTGAG |
| TRIML2 | TRIML2_R | TGAGCTGTTTCAGGATCCAA |
| GADD45 | GADD45A_F | GCTGGTGACGAATCCACATT |
| GADD45 | GADD45A_R | CATCACCGTTCAGGGAGATTA |
| RND3 | RND3_F | GGCCAGTTTTGAAATCGACA |
| RND3 | RND3_R | TCAAAGCAAATCAGCACAGC |
| HECW1 | HECW1_F | GGAATAACACTGAGTACCGG |
| HECW1 | HECW1_R | TGTTCCCGTGACAAACTGCA |
| BIRC3 | BIRC3_F | CCAAGTGGTTTCCAAGGTGT |
| BIRC3 | BIRC3_R | TTTTCATCTCCTGGGCTGTC |
| XBP1 | XBP1_F | TTACGAGAGAAAACTCATGGCC |
| XBP1 | XBP1_R | GGGTCCAAGTTGTCCAGAATGC |
| RNF8 | RNF8_F | AGGTCAAGGGCTTGTTCTGA |
| RNF8 | RNF8_R | AAGAACCCCACACTCCTACT |
| BARD1 | BARD1_F | AGAACAGCTGTTGCCAAAGC |
| BARD1 | BARD1_R | GCTTTCTACTGAGGATCTGG |
