# Supplementary material for: Suppression of BRCA1 sensitizes cells to proteasome inhibitors
Source: Cell Death Dis. 2014 Dec 18;5(12):e1580–. doi: 10.1038/cddis.2014.537 (PMC4649846; doi:10.1038/cddis.2014.537)
Supplement: Supplementary information [file cddis2014537x8.doc]

**Suppression of BRCA1 sensitizes cells to proteasome inhibitors.**

Yuexi Gu^1^, Peter Bouwman^2^, Dario Greco^3^, Jani Saarela^1^, Bhagwan Yadav^1^, Jos Jonkers^2^, and Sergey G. Kuznetsov^1*^

**Supplementary information**

**Supplementary Figure S1.** Proteasome inhibitors inhibit growth of BRCA1- but not BRCA2-deficient cells. (**a**) Knockdown of BRCA1, but not BRCA2, sensitizes MDA-MB-231 (left), HeLa (middle) and U2OS (right) cells to a proteasome inhibitor carfilzomib. Cell viability was measured in 96-well plates using CellTitre-Blue reagent to detect viable cells after 4 days of incubation with indicated doses of bortezomib. All treatments were performed in 4 replicas (N=4). (**b**) Same as (**a**) with two independent siRNAs for BRCA1 and BRCA2 in U2OS cells; N=4. (**c−e**) qRT-PCR demonstrating the efficiency of siRNAs targeting BRCA1 (**c**), BARD1 (**d**), or RNF8 (**e**) in HeLa cells. siControl, non-targeting negative control siRNA; siBRCA1, siRNAs targeting BRCA1. Error bars indicate standard deviations.

**Supplementary Figure S2.** TP53 is not essential for induction of apoptosis in BRCA1-depleted cells treated with bortezomib. Depletion of BRCA1 in HeLa (**a**) and U2OS (**b**) cells results in induction of p21 and cPARP after bortezomib treatment. Although a concomitant knockdown of p53 reduced induction of p21, apoptosis was not affected.

**Supplementary Figure S3.** Microarray analysis identified genes specifically up- or downregulated in response to both BRCA1 knockdown and bortezomib treatment. Heat maps show top differentially expressed genes at the gene (**a**) and transcript (**b**) levels in HeLa and U2OS cells treated with siRNA and indicated doses of bortezomib for 8 hours. Only protein-coding genes and miRNAs were analysed. Ratios of gene expression before and after bortezomib treatment were calculated for each siRNA transfection, and genes were ranked according to the difference between these ratios for siBRCA1- and siCtrl samples. Genes with inconsistent gene expression between HeLa and U2OS cell lines were excluded. Transcripts are designated according to Ensembl nomenclature.

**Supplementary Figure S4.** Death Receptor signaling uderlies toxicity of bortezomib in BRCA1-deficient cells. (**a**) autographs of the Apoptosis Protein Array (Cell Signaling) used to identify apoptotic pathways underlying toxicity of bortezomib in BRCA1-deficient HeLa cells. Treatments are indicated above and on the left side. Numbers and letters indicate coordinates identifying each antibody. (**b**), apoptotic proteins corresponding to each spot on the array identified by unique coordinates. (**c**), bar graph showing spot densities for selected proteins. Notice that TRAIL R1/DR4 is induced to the highest level when treatment with siBRCA1 and bortezomib are combined.

**Supplementary Figure S5.** Multiple genes are involved in regulation of apoptosis induced by bortezomib. 2-4 siRNAs targeting DUSP5 (**a**), CHEK2 (**b**), DR4 (**c**), Cyclin A (**d**), TNFRSF9 (**e**), ERN1 (**f**), TRIML2 (**g**), DAPK2 (**h**), CtIP/RBBP8 (**i**), BIRC3 (**i**), or HECW1 (**k**) were tested for the ability to prevent apoptosis in HeLa cells depleted for BRCA1 and treated with 20 nM bortezomib for 20 hours. Apoptosis was measured by the amount of cleaved PARP. GAPDH served as a protein loading control. Asteriscs indicate siRNAs used in main figures.

**Supplementary Table S1.** siRNAs and their target sequences.

**Supplementary Table S2.** Results of a high throughput drug screening on MDA-MB-231 cells depleted for BRCA1. Viability of control (siControl) and BRCA1-depleted (siBRCA1) cells was compared after 72h incubation with drugs at 5 concentrations ranging from 1 nM to 10 μM as measured using the CellTiter-Blue reagent. Dose response curves are shown in columns E and H, and 50% inhibitory concentrations (IC50) and drug sensitivity scores (DSS) for each cell line for each drug are given in columns C and F, and D and G, respectively. Drugs are arranged according to their difference in DSS (delta DSS, column I) with those selectively toxic against BRCA1-depleted cells at the top, toxic against control cells at the bottom, and those showing no preference between cell lines in the middle. Known molecular targets for each drugs are listed in column K.

**Supplementary Table S3.** PCR primer sequences.
